# Vascular variations encountered during laparoscopic surgery for transverse colon, splenic flexure, and descending colon cancer: a retrospective cohort study

**DOI:** 10.1186/s12893-022-01603-1

**Published:** 2022-05-10

**Authors:** Toshihiro Nakao, Mitsuo Shimada, Kozo Yoshikawa, Takuya Tokunaga, Masaaki Nishi, Hideya Kashihara, Chie Takasu, Yuma Wada, Toshiaki Yoshimoto, Syoko Yamashita, Yosuke Iwakawa

**Affiliations:** Department of Digestive and Transplant Surgery, Tokushima University Hospital, 3-18-15 Kuramoto-cho, Tokushima City, Tokushima 7708503 Japan

**Keywords:** Vascular variation, Transverse colon, Splenic flexure, Descending colon, Middle colic artery, Left colic artery, Middle colic vein, Jejunal vein

## Abstract

**Background:**

Laparoscopic surgery for cancer located in the transverse colon or splenic flexure is difficult because of vascular variability in this region and adjacent vital organs such as the pancreas, spleen, and duodenum.

**Methods:**

This retrospective cohort study involved 51 patients who underwent laparoscopic surgery for colon cancer at Tokushima University Hospital from July 2015 to December 2020. Variations of the middle colic artery (MCA), left colic artery (LCA), middle colic vein (MCV), and first jejunal vein (FJV) and short-term outcomes of laparoscopic surgery in patients with each vascular variation were evaluated.

**Results:**

Variations of the MCA, LCA, MCV, and FJV were classified into four, three, five, and three patterns, respectively. The short-term outcomes of laparoscopic surgery for transverse colon cancer in patients with MCA variations and those with FJV variations were evaluated, and no significant difference was found in the operation time, blood loss, postoperative complication rate, time from surgery to start of dietary intake, or time from surgery to discharge among the different variations. Additionally, no significant differences were found in the short-term outcomes of laparoscopic surgery for descending colon cancer in patients with LCA variations.

**Conclusion:**

Preoperative assessment of vascular variations may contribute to the stability of short-term outcomes of laparoscopic surgery for transverse colon, splenic flexure, and descending colon cancer.

## Background

Laparoscopic surgery for colorectal cancer has been performed worldwide in recent years. Several randomized controlled studies that compared laparoscopic surgery with open surgery for colorectal cancer showed the feasibility and safety of laparoscopic surgery [1–5]. However, these studies excluded transverse colon cancer because of the difficulty of laparoscopic surgery in such cases. Laparoscopic surgery for cancer located in the transverse colon or splenic flexure is more difficult than that for cancer located at other sites [6]. This difficulty is attributed to the vascular variability in this region and adjacent vital organs such as the pancreas, spleen, and duodenum [7]. Some studies have focused on the vascular anatomy of the middle colic artery (MCA) or left colic artery (LCA) and anatomically difficult vascular patterns in laparoscopic surgery [8, 9]. To our knowledge, however, no study has been performed to evaluate multiple vascular variations and short-term outcomes of laparoscopic surgery for each variation. Evaluation of the MCA and LCA together is important in the surgical treatment of splenic flexure cancer because dissection of both arteries is sometimes needed. The present study was performed to identify vascular variations and difficult vascular patterns in patients undergoing laparoscopic surgery for cancer located between the transverse colon and descending colon and to evaluate the short-term outcomes of laparoscopic surgery in patients with each vascular variation.

## Patients and methods

### Patients

This retrospective cohort study involved 51 patients who underwent laparoscopic surgery for colon cancer at Tokushima University Hospital from July 2015 to December 2020. The inclusion criteria were an age of > 20 years and treatment by laparoscopic surgery for cancer fed by the MCA and/or LCA. The exclusion criteria were the presence of other malignant diseases and simultaneous surgical treatment at other sites.

### Definitions of arteries and veins

We defined the right branch of the MCA (Rt-MCA) as the artery running toward the hepatic flexure and the right side of the transverse colon. The left branch of the MCA (Lt-MCA) was defined as the artery running toward the splenic flexure and the left side of the transverse colon from the common trunk of the MCA or the superior mesenteric artery (SMA) at the distal side of the first jejunal artery. The accessory MCA (acMCA) was defined as the artery running toward the splenic flexure and the left side of the transverse colon from the SMA at the proximal side of the first jejunal artery.

The LCA was defined as the artery running toward the splenic flexure and descending colon from the inferior mesenteric artery (IMA). The middle colic vein (MCV) was defined as the vein that drained from the transverse colon. The first jejunal vein (FJV) was defined as the first major branch of the SMV draining from the oral side of the jejunum.

### Definitions of the anatomical vessel variations

**Fig. 1:**
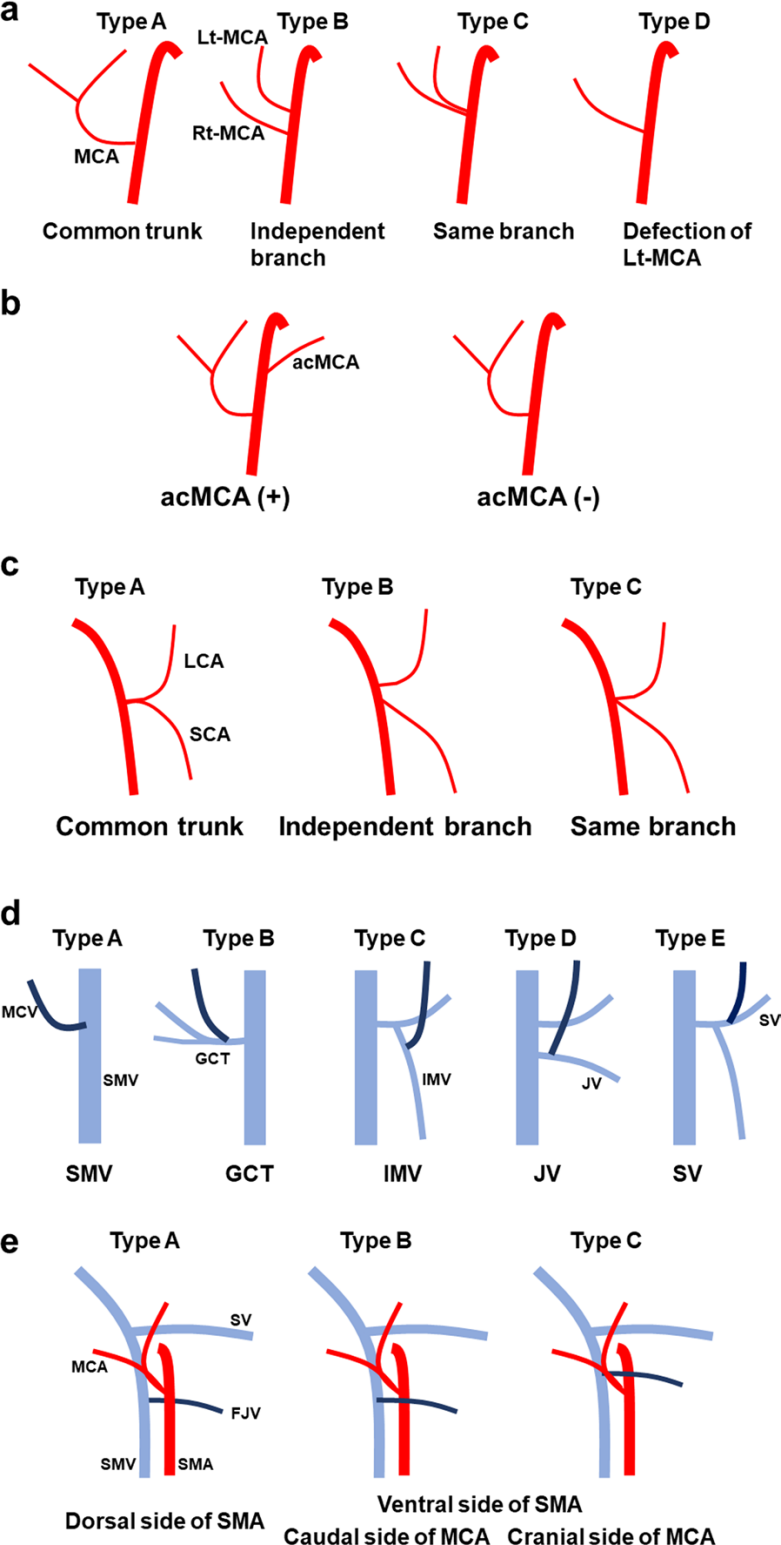
Vascular variations. Variations of the MCA were classified into four patterns (**a**). The acMCA was present in 33.3% of patients (**b**). Variations of the LCA were classified into three patterns (**c**). Variations of the MCV were classified into five patterns (**d**). Variations of the FJV were classified into three patterns (**e**)

Variations of the MCA were classified into four patterns (Fig. 1a). In the first pattern (Type A), the Rt-MCA and Lt-MCA had a common trunk. In the second pattern (Type B), the Rt-MCA and Lt-MCA independently branched from the SMA. In the third pattern (Type C), the Rt-MCA and Lt-MCA each branched from the same point in the SMA. In the fourth pattern (Type D), the Lt-MCA was deficient. Those with SMA between the Rt-MCA and Lt-MCA branches, or no part for ligation to the common trunk were classified as Type B. The artery feeding the ascending colon other than the Rt-MCA was designated as the right colic artery. In the case of no existence of the right colic artery, the artery feeding the anal side of the ascending colon or the right side of the transverse colon was designated as Rt-MCA.

Variations of the LCA were classified into three patterns (Fig. 1c). In the first pattern (Type A), the LCA and sigmoid colic artery (SCA) had a common trunk. In the second pattern (Type B), the LCA and SCA independently branched from the IMA. In the third pattern (Type C), the LCA and SCA each branched from the same point in the IMA. Those with IMA between the LCA and SCA, or no part for ligation to the common trunk were classified as Type B.

Variations of the MCV were classified into five patterns (Fig. 1d). In the first pattern (Type A), the MCV branched from the superior mesenteric vein (SMV). In the second pattern (Type B), the MCV branched from the gastrocolic trunk. In the third pattern (Type C), the MCV branched from the IMV. In the fourth pattern (Type D), the MCV branched from the jejunal vein. In the fifth pattern (Type E), the MCV branched from the splenic vein.

Variations of the FJV were classified into three patterns according to a previous report [10] (Fig. 1e). In the first pattern (Type A), the FJV ran behind the SMV. In the second pattern (Type B), the FJV ran in front of the SMA, and the MCA originated cephalad to the FJV. In the third pattern (Type C), the FJV ran in front of the SMA, and the MCA originated caudad to the FJV.

### Definitions of tumor locations

A tumor that was located on the transverse colon and fed by the MCA was defined as transverse colon cancer. A tumor that was located on the descending colon and fed by the LCA was defined as descending colon cancer. A tumor that was located on the transverse colon near the splenic flexure or descending colon near the splenic flexure and fed by both the MCA and LCA was defined as splenic flexure cancer.

### Definition of postoperative complication

Postoperative complications were defined as any deviation from the normal postoperative course according to the Clavien–Dindo classification [11].

### Evaluation of vascular anatomical variations

**Fig. 2:**
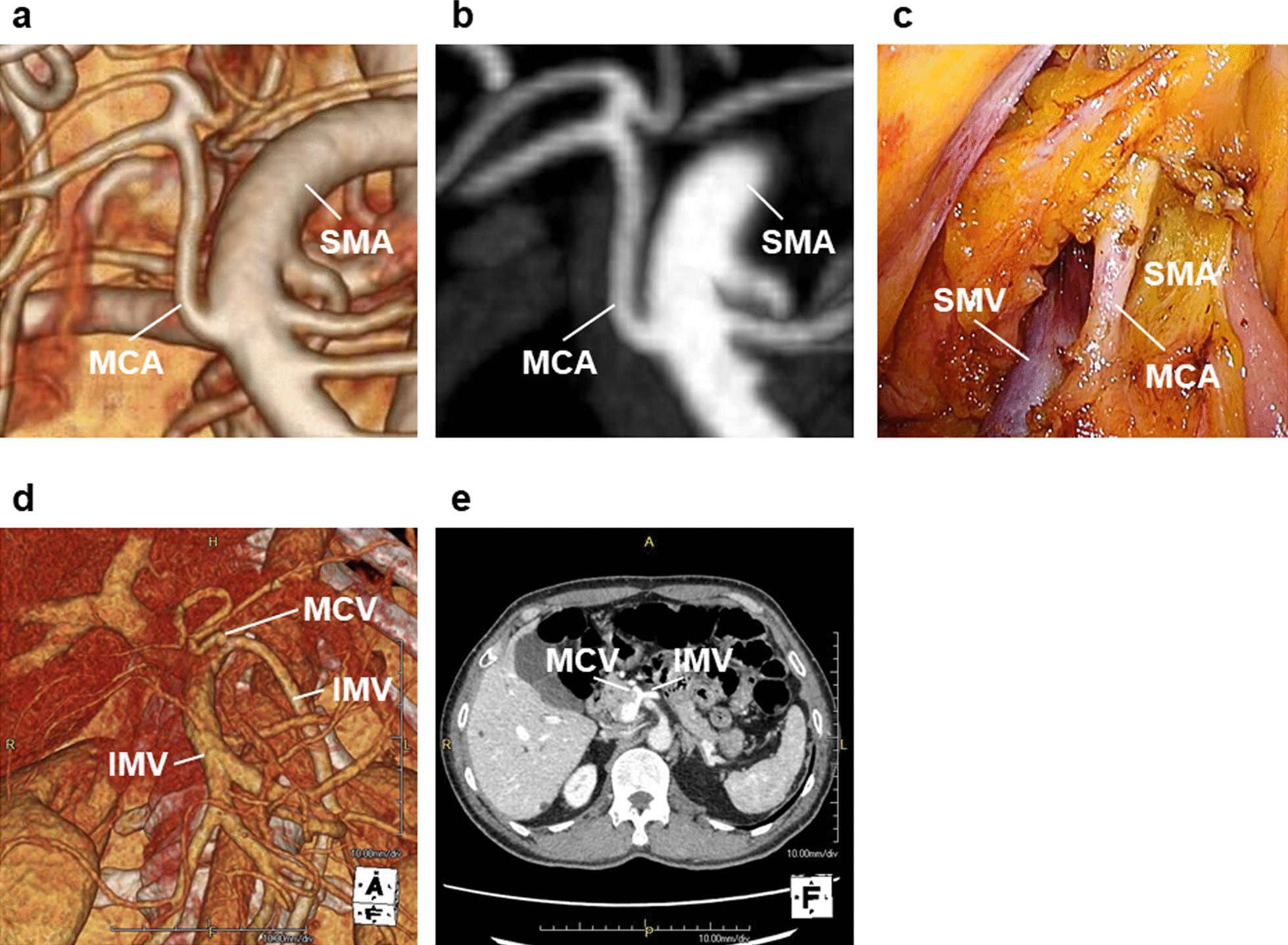
CT and operation findings. **a** 3D CT of MCA Type A. **b** MIP (Maximum Intensity Projection) CT of MCA Type. **c** The operation field of MCA Type A. **d** 3D CT of MCV Type C. There was no MCV on the right side of SMV. **e** CT of MCV Type C

Vascular anatomical variations were evaluated by contrast-enhanced computed tomography (CT) within 1 month before surgery (Fig. 2a, b, d and e). One doctor at the department of surgery who was blinded to the clinical data evaluated the vascular anatomy.

### CT scan protocol

A 320-detector row CT scanner (Aquilion ONE™ / GENESIS Edition, Canon Medical Systems, Tokyo, Japan) was used. Scanning parameters were as follows: tube voltage, 120 kV; tube current, auto exposure control; tube rotation speed, 0.5 per second; slice thickness, 0.5 mm; reconstruction kernel, FC14; image reconstruction interval, 0.5 mm; helical pitch, 129 and interpolation method, 180 degrees interpolation method. Dynamic contrast-enhanced CT was performed using a bolus tracking technique, where a region of interest was placed on the abdominal aorta and the trigger threshold inside the region of interest was set at 120 HU. The first phase scan was started 8 s after the threshold was achieved following the administration of contrast material. The second phase image was obtained 15 s after the first arterial phase. The 3D images were reconstructed using Intuition™ Thin Client version 4.4.13.P7 software (TeraRecon, Tokyo, Japan).

### Surgical procedure

Laparoscopic colectomy for tumors located between the transverse colon and descending colon was performed for all patients in this study.

For tumors fed by the MCA, the MCA or branch of the MCA and the MCV were divided by a caudal approach. When another feeding artery was present, it was also divided. The hepatic flexure, splenic flexure, or both were then mobilized depending on the tumor location.

For tumors fed by the LCA, the LCA and inferior mesenteric vein (IMV) were divided by a medial approach. The splenic flexure was then mobilized by a lateral and cranial approach.

For tumors fed by both the MCA and LCA, the LCA and IMV were divided by a medial approach. The splenic flexure was then mobilized by a lateral and cranial approach. Finally, the MCA or branch of the MCA and the MCV were divided by a caudal approach.

The mesocolon was divided, and reconstruction was performed by extracorporeal functional end-to-end anastomosis in all patients.

The definitions of the operation were following; right hemicolectomy: the operation that divides ileocecal artery and Rt-MCA, left hemicolectomy: the operation that divides Lt-MCA and LCA, transverse colectomy: the operation that divides MCA or branch of MCA, not ileac artery or LCA, and descending colectomy: the operation that divides LCA, not MCA.

### Short-term outcomes of laparoscopic surgery

The operation time, blood loss, postoperative complications, time from surgery to start of dietary intake, and time from surgery to discharge were evaluated.

### Statistical analysis

All statistical analyses were performed with EZR (Version 1.54) (Saitama Medical Center, Jichi Medical University, Saitama, Japan), a graphical user interface for R (Version 4.03) (R Foundation for Statistical Computing, Vienna, Austria) [12]. EZR is a modified version of R commander (Version 2.7-1) designed to add statistical functions frequently used in biostatistics. Categorical variables were analyzed with Fisher’s exact test. Continuous variables were analyzed with the Kruskal–Wallis test. Multivariate analysis was carried out based on the Cox proportional hazard regression model. A *p* value of < 0.05 was considered statistically significant.

## Results

### Patient characteristics

In total, 51 patients were evaluated in this study. The characteristics of all patients and the results of a comparison among patients with transverse colon cancer, descending colon cancer, and splenic flexure cancer are summarized in Table 1.

**Table 1:** Patient’s characteristics

	Total (n = 51)	Transverse colon (n = 32)	Descending colon (n = 17)	Splenic flexure (n = 2)	*p*
Age, years	72 (36–92)	74 (54–92)	70 (36–81)	64 (62–66)	0.083
Sex					
Male	25	15	9	1	0.082
Female	26	17	8	1	
BMI, kg/m^2^	22.5 (16.2–33.3)	22.5 (33.1–16.2)	22.8 (18.8–33.3)	25.8 (22.0–29.6)	0.611
ASA-PS					
1	9	4	5	0	0.366
2	36	25	9	2	
3	5	2	3	0	
4	1	1	0	0	
Tumor size, cm	3.5 (0.5–13.5)	3.3 (0.5–11.8)	3.6 (0.8–13.5)	1.1 (1.0–1.2)	0.182
T					
Tis	4	3	1	0	0.246
T1	13	8	3	2	
T2	6	4	2	0	
T3	9	3	6	0	
T4	19	14	5	0	
N					
N0	34	21	11	2	0.919
N1	12	7	5	0	
N2	5	4	1	0	
M					
M0	46	30	14	2	0.453
M1	5	2	3	0	
Stage					
0	4	3	1	0	0.803
I	19	12	5	2	
II	12	7	5	0	
III	11	8	3	0	
IV	5	2	3	0	
Operation					
Right hemicolectomy	2	2	0	0	
Left hemicolectomy	2	0	0	2	
Transverse colectomy	30	30	0	0	
Descending colectomy	17	0	17	0	

*ASA-PS* American Society of Anesthesiologists physical status, *BMI* body mass index

### Frequencies of the vessel variations

The frequencies of the MCA variations were as follows: Type A, 72.6% (n = 37); Type B, 15.7% (n = 8); Type C, 2.0% (n = 1); and Type D, 9.8% (n = 5). The acMCA (Fig. 1b) was present in 33.3% of patients (n = 17). The left side of the transverse colon was fed by the acMCA in Type D. The frequencies of the LCA variations were as follows: Type A, 27.5% (n = 14); Type B, 45.0% (n = 23); and Type C, 27.5% (n = 14). The frequencies of the MCV variations were as follows: Type A, 70.6% (n = 36); Type B, 21.5% (n = 11); Type C, 2.0% (n = 1); Type D, 2.0% (n = 1); and Type E, 3.9% (n = 2). The frequencies of the FJV variations were as follows: Type A, 76.5% (n = 39); Type B, 15.7% (n = 8); and Type C, 7.8% (n = 4). The frequencies of all vessel variations were summarized in Table 2.

**Table 2:** The frequencies of vessel variations

MCA	
Type A	72.6% (n = 37)
Type B	15.7% (n = 8)
Type C	2.0% (n = 1)
Type D	9.8% (n = 5)
acMCA	
(+)	33.3% (n = 17)
(−)	66.7% (n = 34)
LCA	
Type A	27.5% (n = 14)
Type B	45.0% (n = 23)
Type C	27.5%(n = 14)
MCV	
Type A	70.6% (n = 36)
Type B	21.5% (n = 11)
Type C	2.0% (n = 1)
Type D	2.0% (n = 1)
Type E	3.9% (n = 2)
FJV	
Type A	76.5% (n = 39)
Type B	15.7% (n = 8)
Type C	7.8% (n = 4)

*MCA* middle colic artery, *acMCA* accessory middle colic artery, *LCA* left colic artery, *MCV* middle colic vein, *FJV* first jejunal vein

### Short-term outcomes in patients with each vascular variation

The short-term outcomes of laparoscopic surgery for transverse colon cancer in patients with MCA variations or FJV variations were evaluated. There was no significant difference in the operation time, blood loss, postoperative complication rate, time from surgery to start of dietary intake, or time from surgery to discharge among patients with different types of MCA variations or those with different types of FJV variations (Tables 3, 4). Additionally, no significant differences were found in the short-term outcomes of laparoscopic surgery for descending colon cancer in patients with LCA variations (Table 5).

**Table 3:** Short-term outcomes in MCA variations

	Type A (n = 24)	Type B (n = 4)	Type C (n = 1)	Type D (n = 3)	*p*
Operation time, min	222.3 ± 66.8	242.3 ± 59.5	150.0	179.3 ± 95.2	0.319
Blood loss, ml	12.3 ± 14.4	6.3 ± 4.8	5	9.7 ± 16.7	0.446
Complication	6 (25.0%)	1 (25.0%)	1 (100%)	0 (0%)	0.397
Dietary intake, day	6.3 ± 5.5	4.5 ± 1.3	4.0	4.0 ± 1.0	0.849
Discharge, day	14.0 ± 9.3	11.3 ± 1.3	28.0	12.0 ± 2.5	0.508

*MCA* middle colic artery

**Table 4:** Short-term outcomes in FJV variations

	Type A (n = 23)	Type B (n = 5)	Type C (n = 4)	*p*
Operation time, min	216,8 ± 72.0	203.0 ± 71.3	247.3 ± 37.8	0.508
Blood loss, ml	9.8 ± 12.3	10.0 ± 11.7	18.8 ± 22.5	0.646
Complication	6 (26.1%)	1 (20.0%)	1 (25.0%)	1.000
Dietary intake, day	5.4 ± 4.1	4.0 ± 0.0	9.8 ± 9.6	0.309
Discharge, day	13.6 ± 8.4	12.8 ± 8.5	17.5 ± 10.5	0.230

*FJV* first jejunal vein

**Table 5:** Short-term outcomes in LCA variations

	Type A (n = 4)	Type B (n = 9)	Type C (n = 4)	*p*
Operation time, min	240.8 ± 60.3	238.0 ± 60.1	194.5 ± 30.8	0.386
Blood loss, ml	26.0 ± 21.2	44.1 ± 43.9	382.5 ± 745.1	0.311
Complication	1 (25.0%)	1 (11.1%)	1 (25.0%)	1.000
Dietary intake, day	4.5 ± 0.6	5.1 ± 3.0	4.0 ± 0.0	0.245
Discharge, day	10.5 ± 4.2	10.3 ± 3.3	9.3 ± 2.4	0.656

*LCA* left colic artery

## Discussion

In this study, we investigated vascular variations, which are important in laparoscopic surgery for transverse colon, splenic flexure, and descending colon cancer. We classified the MCA into four patterns. The most frequent pattern, Type A, is considered to require careful attention to prevent injury when dissecting lymph nodes around the root of the MCA with preservation of one branch of the MCA. The acMCA was present in 33.3% of patients. The pattern in which the acMCA is present is considered to require more attention to the risk of pancreatic injury during vascular treatment than the other patterns. We classified the LCA into three patterns. Zhang et al. reported that the rates of our Type A, Type B and Type C were 8.5%, 59.5% and 29.2%, respectively [13]. Our result of the rate of Type A was higher and the rate of Type B was lower than their result, however, our results of the rate of type C and the total rate of Type A and Type B approximated their result. These differences may be due to systematic errors in classification. Type A, which accounted for 27.5% of the total, is considered to have a higher risk of vascular injury than the other types when dissecting lymph nodes around the root of the IMA and treating the LCA with preservation of the SCA. Patroni et al. reported that the LCA can be damaged during the division of the IMA by the low-tie technique in patients with Type A or Type C [9]. The low-tie technique is a procedure that preserves the LCA and divides the IMA. We classified the MCV into five patterns. Types C, D, and E are rare patterns but should be known to prevent injury. Maki et al. reported that the frequency of Type D was only 0.6%; however, knowing this pattern may be helpful to avoid incorrect dissection of the gastrocolic trunk during transverse colectomy [14]. We classified the FJV into three patterns according to a report by Hamada et al. [10]. The authors suggested that Type B and C patterns must be given the greatest attention during MCA ligation to prevent FJV injury.

In this study, we also compared the short-term outcomes of laparoscopic surgery for each vascular variation and found no difference among them. We perform a detailed preoperative evaluation of vascularization by three-dimensional contrast-enhanced CT for all patients. Mari et al. reported that preoperative and intraoperative evaluation of mesenteric vessels by three-dimensional CT angiography decreased the operation time, episodes of difficult identification of correct anatomy, and the incidence of intraoperative and postoperative complications [15]. In this study, the detailed preoperative and intraoperative vascular assessment may be one of the reasons why there were no significant differences in short-term outcomes.

This was a retrospective, single-institution study. Therefore, the sample size was small, and there was a possibility of selection bias. A cohort study with multicenter data is required to further confirm our findings. Also, there are some limitations of the CT scan. Dynamic contrast-enhanced CT could not be performed for the patients who have an allergy to contrast medium or who have severe renal dysfunction. The description of very small vessels is not always sufficient because of the limitation of spatial resolution. The finding of the CT scan differs slightly from the findings of the real operation field because of the factors such as pneumoperitoneum, traction with forceps and gravity on surgical position (Fig. 2a–c).

In conclusion, we analyzed and classified vascular variations in patients undergoing laparoscopic surgery for transverse colon, splenic flexure, and descending colon cancer. Additionally, we compared the short-term outcomes of laparoscopic surgery in patients with each vascular variation, and no significant differences were found. Preoperative assessment of vascular variations may contribute to the stability of short-term outcomes.

## Data Availability

All data generated or analyzed during this study are included in this published article and its additional information files.
